# Reconstructive ossiculoplasty options in primary cholesteatoma surgeries with an intact stapes

**DOI:** 10.1007/s00405-023-08147-x

**Published:** 2023-08-05

**Authors:** Frederic Acke, Filip Kostadinov, Christoph Schlegel, Thomas Linder

**Affiliations:** 1 Department of Otorhinolaryngology, Head and Neck Surgery, Luzerner Kantonsspital, Lucerne, Switzerland; 2 Department of Otorhinolaryngology, Ghent University/Ghent University Hospital, Ghent, Belgium; 3 University of Luzern, Lucerne, Switzerland

**Keywords:** Cholesteatoma, Hearing, Air–bone gap, Incus interposition, Stapes augmentation

## Abstract

**Purpose:**

In primary cholesteatoma patients, incus destruction with an intact and mobile stapes is a frequent finding. Different techniques have been described to restore the ossicular chain, including incus interposition, stapes augmentation and type III tympanoplasty. Controversy about postoperative hearing results in open versus closed surgical techniques exist.

**Methods:**

We performed a retrospective analysis of clinical, surgical and audiometric data of patients with primary cholesteatoma surgery operated between 2010 and 2020, and a mobile stapes and one-stage ossicular reconstruction. Pre- and post-operative audiograms were compared for the different surgical groups, mainly focusing on postoperative air–bone gap. Mastoid pneumatization and ventilation was also considered.

**Results:**

The mean postoperative air–bone gap (0.5–4 kHz) of the 126 included patients was 20 dB. Hearing after type III tympanoplasty (26 dB) was worse than incus interposition (19 dB) and stapes augmentation (20 dB). Hearing after an open (23 dB) versus closed (19 dB) surgical technique was significantly different. No improvement in air–bone gap was observed for the higher frequencies.

**Conclusion:**

A residual postoperative air–bone gap should be considered after primary cholesteatoma surgery with intact and mobile stapes. Incus interposition in closed cavity operation is the optimal situation, but open cavity surgery should not be avoided because of hearing. Extent of the disease is prioritized and poorer ventilation before and after surgery may affect postoperative hearing.

## Introduction

Primary acquired cholesteatomas in children and adults might result in conductive hearing loss due to ossicular chain involvement. Erosion of the long process of the incus is most frequently observed [1]. The integrity and mobility of the stapes cannot be determined preoperatively, since even high-resolution CT or conebeam CT scans are often difficult to interpret due to obliteration of the oval window niche by cholesteatoma or granulation tissue [2]. To find an intact and mobile stapes offers ideal reconstructive options, including incus interposition with autologous or titan incus, stapes augmentation with autologous incus, cartilage or partial ossicular replacement prosthesis (PORP), and a type III tympanoplasty in case of a lowered tympanic membrane, mainly in open mastoido-epitympanectomy (oMET).

There are two main debates in hearing function after cholesteatoma surgery currently discussed: do open cavity surgeries provide less good hearing outcomes compared with closed cavity techniques and should ossiculoplasties be performed immediately or staged? Regarding the first, poor middle ear ventilation and more extensive preoperative pathology might impact postoperative hearing in open cavity surgery [3]. However, studies do not unequivocally report worse hearing outcomes after oMET compared to closed cavity surgery, even without considering the above-mentioned factors necessitating oMET surgery. Regarding the decision about concurrent or staged ossicular chain reconstruction in cholesteatoma surgery, a shift has been observed in the last decade. Previously, staging cholesteatoma surgeries was also intended to exclude residual disease at the second stage surgery. Nowadays, observation by non-EPI-diffusion MRI scans in closed cavity and otoscopically in open cavity surgeries is possible. Since more than 12 years, ossicular reconstruction at first surgery is attempted at our center. This is increasingly supported by literature results showing that staged cholesteatoma surgery seems only advantageous in patients with most severe disease and uncertainty about the complete removal of cholesteatoma [4, 5].

The aim of this study was the evaluation of hearing outcome in primary one-stage cholesteatoma surgery for children and adults in the presence of an intact and mobile stapes, a frequent finding at surgery. By including open and closed surgical techniques, we further intend to compare these, considering eventual modifiers such as the ossicular reconstruction method and the pneumatization/ventilation status of the mastoid, which are included in the ChOLE classification [6].

## Methods

### Surgery

All surgeries were primary surgeries for cholesteatoma. Patients with cholesteatoma extension beyond the epitympanum into the mastoid and poor pneumatization and ventilation as determined on preoperative CT scans were preferably scheduled for an open cavity (oMET) technique following the surgical guidelines of Fisch, May and Linder [7] irrespective of the patient’s age. The posterior canal wall was lowered to the level of the mastoid facial nerve segment; the peri- and supralabyrinthine as well as all mastoid air cells were thoroughly exenterated. The malleus head and incus were always removed and the resected posterior part of the tympanic membrane reconstructed with temporalis fascia. The mastoid tip was either drilled away or completely removed along digastric muscle and the mastoid was partially obliterated using an occipital myosubcutaneous and periosteal flap. The reconstructed drum was lowered onto the posterior canal wall at the level of the mastoid facial nerve. The anterior tympanic membrane angle was kept intact to avoid any blunting. In a closed cavity setting, a circumferential canalplasty allowed full access to the middle ear. The mastoid was opened, leaving the posterior canal wall intact, and was left aerated towards the epitympanum and middle ear without any obliteration.

Ossiculoplasty in either oMET or closed MET was the primary intention at surgery. As all included patients had an intact and mobile stapes, an incus interposition was performed in case of preserved and optimally located malleus handle. In case of a missing malleus or too far anteriorly positioned malleus handle, a stapes augmentation towards the reconstructed tympanic membrane was achieved. Mainly in cases of oMET and an ideally lowered tympanic membrane onto the stapes head, a primary type III tympanoplasty was attempted. Any cartilage placed on top of the stapes was also considered as a stapes augmentation. Immediately after surgery, all patients were entered into a prospective database by coding and a surgeon’s drawing of the pre- and intraoperative findings, mandatory for documentation.

### Data collection

Patients were selected if they met the following inclusion criteria: primary surgery for middle ear cholesteatoma between 01/01/2010 and 31/12/2020, surgery performed by the authors T.L. or C.S. using the same surgical techniques, intact and mobile stapes at the end of the surgery, direct ossicular reconstruction (incus interposition, stapes augmentation or type III reconstruction), and the availability of a pre- and postoperative audiogram with air conduction (AC) and bone conduction (BC) thresholds. Data were extracted from the ENTstatistics database (version 5.1.5.3457, Innoforce Est., Liechtenstein) where clinical, surgical and audiological data of all otology patients including surgical drawings are collected. The following data were extracted: gender, age at surgery, side of surgery, type of surgery, type and material of ossicular reconstruction, status of mastoid pneumatization and ventilation according to the ChOLE classification [6], AC and BC thresholds of the preoperative audiometry closest to the operation date, AC and BC thresholds and timing of the most recent postoperative audiometry, and clinical outcome (recurrent or residual middle ear cholesteatoma on otomicroscopy or MRI, other otomicroscopic anomalies). The coded surgeries were double-checked using the surgeon’s drawings; inconsistent files were excluded. In cases of revision surgeries due to cholesteatoma recidivism or planned further ossiculoplasty because of a bad hearing result, the postoperative audiogram was identified as the one before eventual revision surgery. The study was approved by EKNZ (Ethikkommission Nordwest- und Zentralschweis (BASEC ID 2019-00914).

### Statistical analysis

Statistical analysis was performed using ENTstatistics (version 5.1.5.3457, Innoforce Est., Ruggell, Liechtenstein) and SPSS Statistics (version 28, IBM Corp, Armonk, NY, USA). The pure tone average (PTA) of four frequencies (the average of hearing thresholds at 500, 1000, 2000 and 4000 Hz) was used as a measure for hearing within the speech frequencies. All hearing thresholds were provided in decibel hearing level (dB). Categorical variables were analyzed using the Chi Square test. The Kolmogorov–Smirnov test was used to determine if continuous variables showed a parametric distribution. Subsequently, the Mann–Whitney *U* test and Kruskal Wallis test were performed to compare categorical with continuous variables with non-parametric distribution. A *p* value < 0.05 was considered statistically significant.

## Results

### Baseline characteristics

**Fig. 1:**
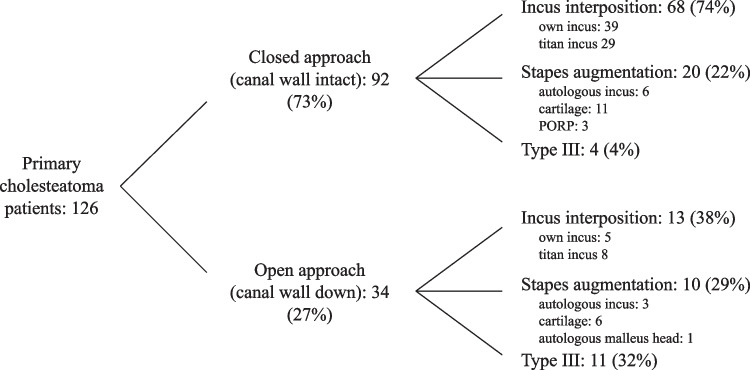
Overview of included patients (surgery between 2010 and 2020), divided into surgical technique, ossicular reconstruction technique and material used for ossicular reconstruction (PORP = partial ossicular replacement prosthesis)

A total of 126 patients out of primary cholesteatoma surgeries between 2010 and 2020 met all the criteria and were included, of whom 42 women (33%) and 84 men (67%). Their mean age at surgery was 40yrs (standard deviation (SD) 21.8yrs); 28 patients (22%) were below 18yrs. Slightly more right than left ears were operated (67 versus 59 respectively). The status of mastoid pneumatization and ventilation, determined by CT scan and/or operative findings, showed moderate to good pneumatization and good ventilation in 42 patients (ChOLE E0, 33%), moderate to good pneumatization but poor ventilation in 41 patients (ChOLE E1, 33%), and poor pneumatization and ventilation in 43 patients (ChOLE E2, 34%). The surgical and ossicular reconstruction techniques are provided in Fig. 1. Open cavity surgeries were performed in 27%, whereas 73% were surgeries with a closed approach.

As expected, a poorer E status (Eustachian tube function) was observed in the open surgical technique compared to the closed approach (oMET: 18% E0, 32% E1, 50% E2; closed approach: 39% E0, 33% E1, 28% E2) (***p* = *0.032***, Chi Square test).

### Preoperative hearing

The mean preoperative pure tone average (PTA, average of 500–4000 Hz) of the total group was 38 dB for AC (SD 17.0 dB), 15 dB for BC (SD 11.8 dB) and 22 dB for ABG (SD 11.0 dB). No statistically significant differences in preoperative hearing were found among the different surgical techniques (closed versus open, *p* = 0.14, *p* = 0.34 and *p* = 0.18 respectively, Mann–Whitney *U* test), nor among the different ossicular reconstruction techniques (incus interposition, stapes augmentation and type III reconstruction, *p* = 0.37, *p* = 0.68 and *p* = 0.23 respectively, Kruskal Wallis test).

### Postoperative hearing

The most recent postoperative audiometry was performed 3.0 years after the operation on average (SD 2.6 year, minimum 2 months, maximum 12 years, 11 patients (8.7%) below 6 months). The mean postoperative PTA for the total group was 36 dB for AC (SD 18.2 dB), 16 dB for BC (SD 12.5 dB) and 20 dB for ABG (SD 20.0 dB). Values for the different surgical and ossicular reconstruction techniques are provided in Table 1. The differences between postoperative and preoperative thresholds were also calculated. The PTA for AC decreased by 2 dB (SD 15.6 dB), the PTA for BC slightly increased by 1 dB (SD 6.7 dB), and the PTA for air–bone gap (ABG) decreased by 2 dB (SD 12.3 dB).

**Table 1:** Postoperative hearing thresholds provided as mean pure tone average (PTA) for air conduction, bone conduction, and air–bone gap, for the different surgical and ossicular reconstruction techniques

	Postoperative PTA air conduction	Postoperative PTA bone conduction	Postoperative PTA air–bone gap
Closed—incus interposition (*n* = 68)	34 dB (SD 19.5 dB)	16 dB (SD 13.2 dB)	18 dB (SD 9.8 dB)
Closed—stapes augmentation (*n* = 20)	37 dB (SD 18.5 dB)	17 dB (SD 11.0 dB)	20 dB (SD 11.7 dB)
Closed—type III (*n* = 4)	35 dB (SD 8.9 dB)	8 dB (SD 6.0 dB)	27 dB (SD 12.1 dB)
Open—incus interposition (*n* = 13)	36 dB (SD 13.1 dB)	12 dB (SD 8.1 dB)	24 dB (SD 12.6 dB)
Open—stapes augmentation (*n* = 10)	37 dB (SD 13.3 dB)	18 dB (SD 11.2 dB)	19 dB (SD 10.7 dB)
Open—type III (*n* = 11)	49 dB (SD 18.0 dB)	23 dB (SD 15.8 dB)	26 dB (SD 10.6 dB)

**Fig. 2:**
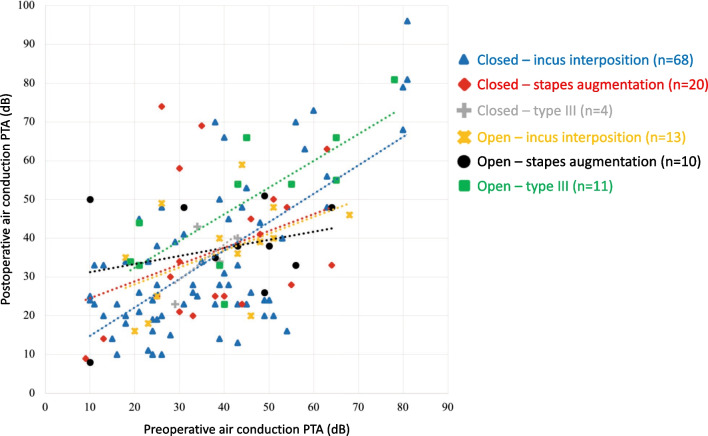
Pre- and post-operative mean air conduction thresholds (PTA) of the total group, differentiated by surgical and ossicular reconstruction technique. A linear trend line is included for the different groups. Optimally, the trend line would be as low and with as little inclination as possible

Of utmost clinical relevance for the patient is the postoperative AC PTA, which is shown in Fig. 2. Of importance regarding treatment effect of ossiculoplasties is the postoperative ABG among the treatment groups, which allowed us to closer look at the variables. The differences among the included surgical and ossicular reconstruction techniques, as well as the ossicular reconstruction material, are provided in Table 2.

**Table 2:** Comparison of different surgical and ossicular reconstruction techniques, as well as ossicular reconstruction material, regarding the mean postoperative air–bone gap PTA

Comparison	Postoperative PTA for air–bone gap	*p* value
Closed approach (*n* = 92)	19 dB (SD 10.4 dB)	***p* = *0.020***
Open approach (*n* = 34)	23 dB (SD 11.4 dB)	
Incus interposition (*n* = 81)	19 dB (SD 10.4 dB)	Incus—stapes: *p* = 0.62 Incus—type III: ***p* = *0.011*** * Stapes—type III: *p* = 0.051
Stapes augmentation (*n* = 30)	20 dB (SD 11.2 dB)	
Type III (*n* = 15)	26 dB (SD 10.6 dB)	
Closed—incus interposition (*n* = 68)	18 dB (SD 9.8 dB)	0.13
Open—incus interposition (*n* = 13)	24 dB (SD 12.6 dB)	
Closed—stapes augmentation (*n* = 20)	20 dB (SD 11.7 dB)	0.71
Open—stapes augmentation (*n* = 10)	19 dB (SD 10.7 dB)	
Closed—incus interposition (*n* = 68)	18 dB (SD 9.8 dB)	0.65
Closed—stapes augmentation (*n* = 20)	20 dB (SD 11.7 dB)	
Open—incus interposition (*n* = 13)	24 dB (SD 12.6 dB)	0.65
Open—stapes augmentation (*n* = 10)	19 dB (SD 10.7 dB)	
Incus interposition—autologous incus (*n* = 44, open + closed)	18 dB (SD 11.4 dB)	0.18
Incus interposition—titan incus (*n* = 37, open + closed)	20 dB (SD 9.1 dB)	
Stapes augmentation—autologous incus (*n* = 9, open + closed)	23 dB (SD 12.6 dB)	0.22
Stapes augmentation—cartilage (*n* = 17, open + closed)	16 dB (SD 7.3 dB)	

*Group comparison: ***p* = *0.037*** (Kruskal Wallis test)

Regarding the status of mastoid pneumatization and ventilation, the mean postoperative ABG was 17 dB (SD 11.4 dB) for E0, 22 dB (SD 10.1 dB) for E1, and 21 dB (SD 10.7 dB) for E2 (*p* = 0.059, Kruskal Wallis test).

**Fig. 3:**
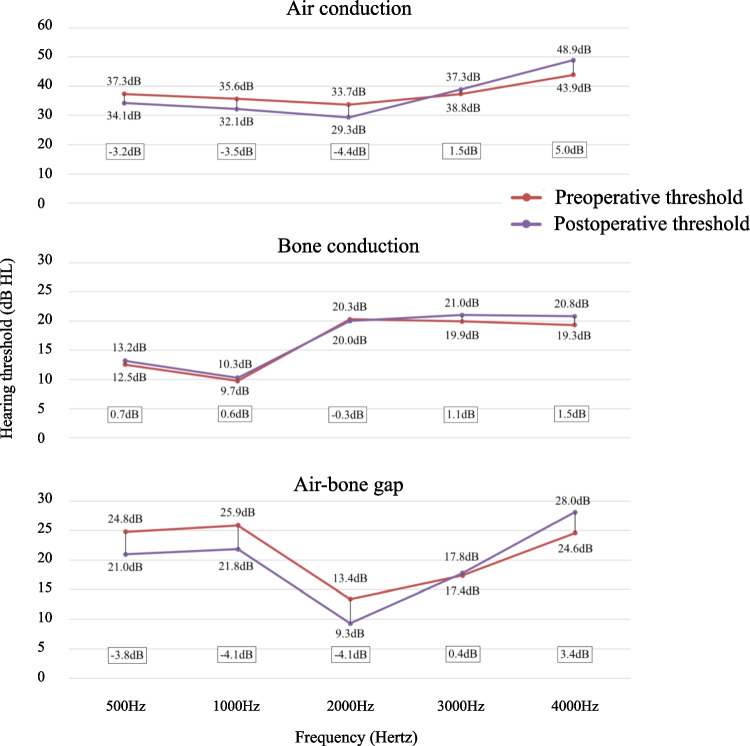
Pre- and post-operative mean air and bone conduction thresholds of the total group, as well as the air bone gap, for the main frequencies between 500 and 4000 Hz

To account for frequency effects, Fig. 3 provides the mean pre- and postoperative AC and BC thresholds of the total group, as well as the ABG, for the main frequencies between 500 and 4000 Hz. A decrease in ABG for 500–2000 Hz was noted in contrast to 3000 and 4000 Hz. This was observed in both closed and open surgical techniques (3000 and 4000 Hz ABG difference of 0 and + 3 dB for closed surgery compared to 0 and + 4 dB for open surgery).

### Best and worst performers

The best performers were defined as those having a postoperative ABG PTA of 15 dB or less (48 patients), whereas the worst performers had a postoperative ABG PTA of 25 dB or more (36 patients). The potential effect of relevant parameters on the outcome is provided in Table 3.

**Table 3:** Relevant parameters related to postoperative hearing categories (best, average, and worst performers)

	Best performers (ABG ≤ 15 dB) *n* = 48	Average performers (ABG 16-24 dB) *n* = 42	Worst performers (ABG ≥ 25 dB) *n* = 36	*p* value
Age at operation	37 years (SD 20.1 years)	40 years (SD 20.8 years)	47 years (SD 23.4 years)	0.16
Operation technique	17% open 83% closed	31% open 69% closed	36% open 64% closed	0.11
Ossicular reconstruction technique	75% incus interposition 21% stapes augmentation 4% type III	57% incus interposition 29% stapes augmentation 14% type 3	58% incus interposition 22% stapes augmentation 19% type III	0.18
Pneumatizationand ventilation status	48% E0 25% E1 27% E2	19% E0 43% E1 38% E2	31% E0 31% E1 39% E2	0.059

The *p* value indicates the comparison of the parameter with the three performer categories (Kruskal Wallis test and Chi Square test); pairwise comparisons were not performed

Clinical follow-up showed several causes for the high ABG in the group of worst performers, including five patients with cholesteatoma recidivism (one recurrent and two residual cholesteatomas, and two with a small inclusion cyst in the external ear canal) and three patients with obvious titan incus prosthesis dislocation or extrusion. The five patients with recidivism underwent revision cholesteatoma surgery, and six other patients eventually underwent revision surgery aiming at improving hearing. Of the latter, five improved whereas in one no reconstruction was performed because of newly discovered tympanosclerosis. Of interest, two out of the three patients with PORP reconstruction, and the patient with malleus head used as stapes augmentation were part of the worst performers.

In the total database, 12/126 patients experienced recidivism (9.5%), of whom 7 with middle ear recurrence, 2 with residual cholesteatoma, and 3 with a small inclusion cyst (2 in the external ear canal and 1 in the tympanic membrane). No statistically significant difference between open and closed surgical techniques was observed. They all underwent revision surgery and do not show recidivism after the second surgery to date.

## Discussion

In cholesteatoma patients, different outcome parameters should be considered, such as recidivism (residual and recurrent cholesteatoma), surgical complications, and hearing. In the current retrospective study, we focused on hearing outcome after cholesteatoma surgery, namely patients with ossicular involvement but an intact and mobile stapes. All included patients underwent primary one-stage ossicular reconstruction. Since the availability of non-EPI-diffusion MRI scans, we do not routinely perform staged surgery anymore in closed cavity surgeries. In this way, we avoid increased hearing loss between the two surgeries and avoid unnecessary second look surgeries. In open cavities, there is no need for MRI follow-up since recidivism is observed clinically. One-stage hearing reconstruction seems to be supported by the rather low number of recidivism (9.5%, including 1.6% residual disease, 5.5% true recurrence, and 2.4% external ear canal or tympanic membrane inclusion cyst). Our results show a mean postoperative ABG of 20 dB (average between 500 and 4000 Hz), slightly better than preoperatively, and no significant difference in ABG after surgery. This confirms the clinical observation that cholesteatoma preoperatively still transmits sound to the intact stapes in cases of incus erosion.

Several parameters have been proposed to impact hearing outcome after cholesteatoma surgery. One of the main controversies is whether open versus closed surgical techniques provide better hearing. We found only a small but significantly worse postoperative ABG in oMET surgeries (23 dB) compared to closed cavity surgeries (19 dB). A higher number of type III tympanoplasties, which showed worse hearing outcome compared to the other reconstruction techniques, in oMET surgeries mainly explains this difference. A larger but not significant ABG in oMET surgery with incus interposition (24 dB) versus stapes augmentation (19 dB) was also noted. Hearing in patients who underwent oMET surgery with stapes augmentation proved in the same range as incus interposition or stapes augmentation in closed cavity surgery though. It is hard to compare open versus closed cavity surgeries in view of hearing outcome, as pre-existent factors influencing postoperative hearing have been detected. Examples are poor middle ear and mastoid ventilation and more advanced disease, which dominate oMET surgeries [3]. We believe a small and shallow middle ear cavity is a negative prognostic factor, as shown in type III tympanoplasties independent of an open or closed surgical technique. Middle ear cavity volume, mastoid pneumatization, the size and state of the antrum, and mucosal state are interrelated [8] and are approximated by the E status of the ChOLE classification [7]. The E status in our study was related in a borderline significant way to hearing outcome, with a better pneumatization/ventilation status associated to a lower postoperative ABG. The pneumatization/ventilation status might influence the choice of surgical technique, which in turn might influence the choice of ossiculoplasty technique, making a formal comparison difficult in absence of surgical technique randomization.

The presence or absence of the malleus handle, rather than the stapes suprastructure, was shown as a significant predictor of postoperative hearing outcome in a meta-analysis of patients with cholesteatoma or non-cholesteatoma chronic otitis media [9], which confirmed earlier observations of Dornhoffer et al. [10]. In our series, no significant difference between incus interposition and stapes augmentation could be observed. Therefore we advise stapes augmentation in case of a too far anteriorly positioned malleus handle.

When comparing ossicular reconstruction material within the groups, the better outcome in stapes augmentation with cartilage (16 dB) compared to autologous incus (23 dB) is apparent, although not statistically significant due to a lower number of patients. Ayache et al. thoroughly reviewed the procedure of cartilage stapes augmentation and compared with other autologous and synthetic materials [11]. They also concluded that cartilage stapes augmentation is a safe and effective procedure with no additional cost, and with hearing results at least as good as with other materials. The difference in incus interposition material, autologous versus titan incus, is less pronounced (18 dB versus 20 dB). A further benefit of cartilage or autologous bone is the fact that no extrusion can be observed.

Another interesting finding is the improvement in ABG after ossicular reconstruction surgery for 500, 1000 and 2000 Hz, but not for 3000 and 4000 Hz. At initiation, we decided to report the PTA of 500–4000 Hz as it covers the human speech recognition frequency range, although many other studies only report the PTA of 500–2000 Hz. The results of the latter are more beneficial, also in our series, but render comparison of absolute values among studies more difficult. We previously reported a postoperative increase in high-frequency ABG, mainly in type III tympanoplasty [12]. Here, a stable ABG at 3 kHz and increase at 4 kHz was noted in both open and closed cavity ossicular reconstructions. It is widely known that, preoperatively, cholesteatoma or granulation tissue might transmit sound, even in a discontinued ossicular chain [13]. Based on our results, ossicular reconstruction seems more effective in transmitting sound at the lower frequencies, but not at 4 kHz. In a temporal bone study with incus removal but intact malleus and stapes, several ossicular reconstruction methods were compared [14]. It was found that the optimal reconstruction is the one connecting stapes head to both the malleus handle and tympanic membrane, although poorer sound transmission in the higher frequencies was found for all conditions. However, gluing the contact between stapes head and prosthesis resulted in better high-frequency transmission [14]. It can thus be hypothesized that the higher ABG at 4 kHz after ossicular reconstruction might be related to a rather loose connection between stapes head and the ossicular reconstruction material. Therefore one might have to come up with a new technique of firmer connection towards the stapes head.

The strengths of this study are the consistency of surgical technique among the two surgeons, the availability of complete pure tone audiometry results and the reporting of 4 kHz enabling us to detect additional findings relevant for the patient. Weaknesses are the retrospective nature of the analysis despite prospective acquisition of data, and the lack of speech discrimination results due to inconsistent collection. Moreover, few patients treated with PORP could be included, whereas at other centers PORPs are more frequently used.

In summary, an average ABG of 20 dB (500–4000 Hz) should be considered in primary cholesteatoma cases with incus removal but intact and mobile stapes after one-stage ossicular reconstruction. Hearing after type III tympanoplasty was worse than after incus interposition or stapes augmentation. No difference in hearing between closed cavity incus interposition or stapes augmentation, and open cavity stapes augmentation was observed. The beneficial effect of ossicular reconstruction seemed limited to the lower and middle frequencies (up to 2 kHz), whereas the higher frequencies did not improve compared to the preoperative audiogram. Selecting the type of surgery between open and closed cavity techniques relies not primarily on the attempted hearing outcome but should have in mind the main surgical goal of complete disease removal. This is supported by our low recidivism rate of less than 10%.

## Data Availability

The data supporting the findings of this study are available from the authors upon reasonable request.
